# BACH1 promotes the progression of esophageal squamous cell carcinoma by inducing the epithelial–mesenchymal transition and angiogenesis

**DOI:** 10.1002/cam4.3884

**Published:** 2021-05-01

**Authors:** Yan Zhao, Jiajia Gao, Xiufeng Xie, Peng Nan, Fang Liu, Yulin Sun, Xiaohang Zhao

**Affiliations:** ^1^ State Key Laboratory of Molecular Oncology, National Cancer Center/National Clinical Research Center for Cancer/Cancer Hospital Chinese Academy of Medical Sciences and Peking Union Medical College Beijing China

**Keywords:** angiogenesis, BACH1, ESCC, metastasis, the EMT

## Abstract

Metastasis to regional lymph nodes or distal organs predicts the progression of the disease and poor prognosis in esophageal squamous cell carcinoma (ESCC). Previous studies demonstrated that BTB and CNC homology 1 (BACH1) participates in various types of tumor metastasis. However, the function of BACH1 in ESCC was rarely reported. The present study demonstrated that BACH1 protein was overexpressed in ESCC tissues compared with paired esophageal epithelial tissues according to immunohistochemical staining (IHC). Higher levels of *BACH1* mRNA were associated with decreased overall survival (OS) and shorter disease‐free survival (DFS) of ESCC patients based on an analysis of The Cancer Genome Atlas (TCGA) datasets. BACH1 significantly enhanced the migration and invasion of ESCC in vitro. Mechanistically, BACH1 promoted the epithelial–mesenchymal transition (EMT) by directly activating the transcription of *CDH2*, *SNAI2*, and *VIM*, as determined by chromatin immunoprecipitation‐quantitative polymerase chain reaction (ChIP‐qPCR). *BACH1* overexpression significantly enhanced *CDH2* promoter activity according to the results of a luciferase assay. The results of subsequent experiments indicated that BACH1 enhanced the growth of tumor xenografts. The density of CD31^+^ blood vessels and the expression of vascular endothelial growth factor C (VEGFC) in tumor xenografts were significantly associated with BACH1 levels according to the results of IHC and immunofluorescence (IF) analyses performed in vivo. Moreover, ChIP‐qPCR analysis demonstrated that the transcriptional activity of *VEGFC* was also upregulated by BACH1. Thus, BACH1 contributes to ESCC metastasis and tumorigenesis by partially facilitating the EMT and angiogenesis, and BACH1 may be a promising therapeutic target or molecular marker in ESCC.

## Graphical abstract

We investigated the functions of BACH1 in esophageal squamous cell carcinoma (ESCC), and our data firstly demonstrated that BACH1 contributes to ESCC metastasis and progression by EMT and angiogenesis. Substantial evidence supports the view that BACH1 triggers EMT‐induced metastasis by activating *CDH2*, *SNAI2*, and *VIM* transcription in ESCC. BACH1 acts as the upstream regulator to activate CDH2 through binding to its promoter and facilitates angiogenesis partially by activating transcription of VEGFC.

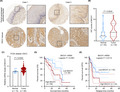

## 1 INTRODUCTION

Esophageal squamous cell carcinoma (ESCC) is the ninth most common malignant cancer and the sixth leading cause of cancer‐related death worldwide.^1^ High propensity for invasion and metastasis of ESCC result in frequent metastases in lymph nodes or distal organs and rapid tumor recurrence in patients. The 5‐year survival rate (20%) of patients with ESCC has remained low in the past decade.^2^ Therefore, investigation of the molecular mechanisms of invasion and metastasis of ESCC may improve our understanding of this disease and lead to the discoveries of novel promising biomarkers and treatment targets.

BACH1 belongs to the cap ‘n’ collar (CNC) transcription factor family. The functional structure of BACH1 includes an N‐terminal BTB/POZ domain that interacts with proteins and a C‐terminal highly conserved leucine zipper (bZIP) structure that binds to DNA.^3^ BACH1 is widely expressed in most mammalian tissues and has been shown to function as a transcription factor by activating or repressing target gene transcription. BACH1 acts as a transcriptional repressor of the antioxidant gene *HMOX*‐*1* in response to oxidative stress under normal conditions in many human cell types.^4^ BACH1 has been reported to participate in the cell cycle, hematopoiesis, angiogenesis, and immunity in various cells and tissues.^3^, ^4^, ^5^, ^6^

Recently, accumulating evidence indicated that BACH1 is highly expressed in many solid tumors. Aberrant expression of BACH1 contributes to tumor metastasis in breast cancer,^7^, ^8^, ^9^ colorectal cancer,^10^, ^11^, ^12^, ^13^ prostate cancer,^14^ ovarian cancer,^15^, ^16^ pancreatic cancer,^17^ osteosarcoma,^18^ and lung cancer.^19^, ^20^ Mechanistically, some of the studies revealed that BACH1 functions as a master transcriptional regulator of cancer progression and metastasis. For example, in *RAS*‐driven pancreatic ductal adenocarcinoma, BACH1 directly represses the expression of the transcription factor *FOXA1*, which is known to activate the expression of *CDH1* encoding E‐cadherin, *CLDN3*, and *CLDN4* to induce the EMT and tumor metastasis.^17^ BACH1 also acts as a critical molecular regulator of breast cancer bone metastasis by activating the transcription of several functional metastatic genes, including *MMP1* and *CXCR4*.^9^ Moreover, in human ovarian cancer, BACH1 promotes ovarian migration by interacting with HMGA2 and enhancing the transcription of EMT genes, including *VIM*, *SNAI1*, and *SNAI2*.^15^ In lung cancer, BACH1 expression stimulates glycolysis by activating *HK2* transcription and triggers glycolysis‐dependent lung cancer metastasis.^19^

Although BACH1 has been reported as a crucial regulator of metastasis in various cancers, its role in ESCC remains unknown. The results of the present study demonstrated that BACH1 protein was overexpressed in ESCC tissues. The data of TCGA RNA sequencing in ESCC indicated that higher levels of *BACH1* mRNA predicted poorer overall survival (OS) and disease‐free survival (DFS). *BACH1* knockdown inhibited the migration and invasion of ESCC cells in vitro and the growth and angiogenesis of xenograft tumors in vivo. Mechanistically, BACH1 activated the EMT by upregulating the transcription of the *CDH2*, *SNAI2*, and *VIM* genes and partially facilitated angiogenesis by activating *VEGFC* transcription. Thus, our results indicated that BACH1 is an oncogenic transcription factor involved in ESCC progression and metastasis.

## 2 MATERIALS AND METHODS

### 2.1 Cell culture

The ESCC cell lines KYSE30, KYSE150, KYSE170, and KYSE510, which were authenticated by DNA fingerprinting analysis, were obtained from Dr. Shimada (Hyogo College of Medicine). Cells were cultured in RPMI 1640 medium (GE Healthcare Life Sciences) supplemented with 10% FBS (HyClone) and antibiotics (100 U/ml penicillin and 100 μg/ml streptomycin) at 37°C with 5% CO_2_ in an incubator.

### 2.2 Immunohistochemistry staining

Tissue microarrays of ESCC, HEsoS105Su01 included 50 tumor tissues and paired esophageal epithelial tissues and ZH‐ESC 77a included 77 tumor tissues, were purchased from Outdo Biotech and Zhuohao Medical Science and Technology (Shanghai, China). Slides of the tissue microarray were incubated with 1:200 diluted BACH1 antibody (#A5393, Abclonal), 1:50 diluted N‐cad antibody (#610920, BD Biosciences), 1:50 diluted vimentin antibody (#A19607, Abclonal), 1:50 diluted Slug antibody (#TA800167, OriGene Technologies), and PBS control overnight at 4°C. Then, tissue arrays were incubated with an anti‐rabbit‐HRP polymer (ZSGB‐Bio) for 20 min, followed by incubation with DAB (ZSGB‐Bio) for 1 min. The results were examined by two independent pathologists who evaluated and scored the slides based on the intensity and extent of the staining observed under a microscope.^21^ A final score between 0 and 12 was calculated by multiplying the positive rate and intensity scores. For BACH1, N‐cadherin, vimentin and Slug, scores of 0–6, 0–3, 0–8, and 0–8 were considered low expression, and other scores were considered high expression.

### 2.3 Immunofluorescence staining

Cells were fixed in 4% paraformaldehyde for 30 min, permeabilized in 0.2% Triton X‐100 for 10 min, and then blocked in 2% bovine serum albumin for 1 h. The fixed cells were incubated overnight with 1:100 diluted BACH1 polyclonal antibodies (#A5393, Abclonal) at 4°C and then incubated with Alexa Fluor 488‐conjugated secondary antibody (1:100, #A11034, Invitrogen) for 1 h. DAPI was used to stain the nuclei. IF images were acquired under a laser confocal microscope (Carl Zeiss).

Paraffin‐embedded tissue sections were fully dewaxed in xylene, antigen‐repaired with citrate buffer at 100°C for 20 min, and incubated with a primary antibody mixture containing rabbit anti‐CD31 (1:100, #ab28364, Abcam) and mouse anti‐CK (Pan) (1:100, #ZM0069, ZSGB‐BIO) overnight at 4°C. Then, Alexa Fluor 594‐conjugated secondary antibody (1:100, #A11037, Invitrogen) and Alexa Fluor 488‐conjugated secondary antibody (1:200, #A11029, Invitrogen) were added and incubated for 1 h. Finally, DAPI was used to stain the nuclei. Images were acquired by a PerkinElmer Vectra Polaris automatic pathological slice scanning system.

### 2.4 Transfection and lentivirus infection

The following siRNA sequences targeting *BACH1* and a negative control were used: BACH1 si1: 5'‐AUAUCAUGGAUACAAUCCAGC‐3’; BACH1 si2: 5'‐GGUCAAAGGACUUUCACAACAUUAA‐3’; and siCtrl: 5'‐UUCUCCGAACGUGUCACGUTT‐3’ (GeneChem). siRNAs and siCtrl were transfected into KYSE30 and KYSE170 cells using Lipofectamine 3000 reagent (Invitrogen) following the manufacturer's instructions.

Full‐length human *BACH1* cDNA (NM_206866.2; CCDS13585.1) was synthesized and inserted into the lentiviral vector pLVX‐IRES‐RFP (ViGene Biosciences, Shandong, China). Short hairpin RNAs^22^ targeting *BACH1* (shBACH1: 5'‐ATATCATGGATACAATCCAGC‐3’) were subcloned into the pLenti‐U6‐GFP‐Puro lentiviral shRNA vector (ViGene Biosciences). The constructed lentiviral vectors were packaged into the viruses in 293 T cells. Then, the harvested and concentrated viruses were added to KYSE150 and KYSE170 cells and cultured for 48 h. The supernatant was replaced with complete culture medium with 1 μg/ml puromycin for selection for 15 days.

### 2.5 Western blotting

Cells were lysed in 0.3% Nonidet P40 buffer (150 mM NaCl and 50 mM Tris‐HCl, pH 7.5) containing protease inhibitor cocktail (Selleck). The cell extract was prepared by centrifugation (12,000 rpm for 15 min). Subsequently, the protein samples (25 μg) were electrophoresed on 10% SDS‐PAGE gels and transferred to polyvinylidene difluoride membranes (Millipore, MA) by electroblotting. The membranes were blocked for 3 h with 5% (w/v) nonfat dry milk in PBS and incubated with primary antibodies at 4°C overnight. Primary antibodies against BACH1 (1:2,000, #A5393, Abclonal), β‐actin (1:5,000, #A2228, Sigma‐Aldrich, MO), E‐cadherin (1:1,000, #3195, CST), N‐cadherin (1:1,000, #13116, CST), Slug (1:1,000, #3879, CST), and vimentin (1:1,000, #5741, CST) were used. The signals were visualized by a MaxiLumin^TM^ WB detection kit (Biokits Tech).

### 2.6 Cell migration and invasion assays

Cell migration and invasion assays were performed as described previously.^22^ Briefly, Transwell 8 μm pore inserts and 24‐well Millicell chambers were used (Costar, MA). The inserts were coated with or without Matrigel (BD Biosciences). A total of 5×10^4^ cells in 100 µL of serum‐free medium were plated into coated or uncoated inserts in triplicate. The lower chambers were supplemented with 600 μl of RPMI 1640 medium with 15% FBS as a chemoattractant. Cells that migrated to the lower layer of the inserts after 36 h were fixed in methanol for 30 min and stained with 0.1% crystal violet for 30 min. The cells were counted in five random fields selected under a microscope.

### 2.7 Wound healing assay

In brief, a total of 1×10^5^ cells were seeded in 24‐well plates and incubated to form a >90% confluent monolayer, which was scratched in a straight line with a 10 μl pipette tip. The cells were cultured in serum‐free RPMI 1640 medium, and the migration was monitored at 0 and 24 h after wounding. The total area between the wound edges was evaluated by ImageJ software to calculate the migration rate.

### 2.8 Quantitative PCR assay

Total RNA was extracted using a Direct‐zol^TM^ RNA MiniPrep kit (Zymo Research). Two micrograms of total RNA was used for reverse transcription with a HiFiScript cDNA synthesis kit (CWBIO) to generate cDNA. QPCR was performed using an ABI QuantStudio DX 96 instrument (Thermo Fisher Scientific) and TB Green^TM^ Premix Ex Taq^TM^ reagent (TaKaRa Bio). The *Ct* values were normalized to the expression of the endogenous housekeeping gene *GAPDH*, and the 2^(−ΔΔCt)^ values were calculated for relative quantification. The reactions were performed in triplicate. The qPCR primers are shown in Table S1.

### 2.9 ChIP qPCR assay

ChIP‐qPCR assays were performed using a ChIP kit and reagents (CST) according to the manufacturer's instructions. First, 5 μg of a BACH1 antibody (#AF5776, R&D Systems) and a corresponding control anti‐IgG antibody were used to immunoprecipitate digested chromatin DNA fragments derived from KYSE150 and KYSE170 cells. Second, qPCR analysis was performed to identify the BACH1‐binding sites within the promoters of *CDH2*, *VIM*, *SNAI2*, and *VEGFC*. The sequences of the ChIP qPCR primers for *CDH2* and negative control were designed in this study, and the sequences of the primers for *HMOX1* (positive control), *VIM*, *SNAI2*, and *VEGFC* were obtained from previous studies.^15^, ^16^, ^17^ The sequences are shown in Table S2.

### 2.10 Dual luciferase reporter assay

For the luciferase assay, a total of 6×10^4^ KYSE150 and KYSE170 cells were plated in 24‐well plates in triplicate. The cells in each well were transfected with 0.1 μg of the promoter‐luciferase plasmid in combination with 0.6 μg of pcDNA3.1 and/or pcDNA3.1‐BACH1. Additionally, the cells were cotransfected with 0.01 μg of pRL‐TK to normalize the transfection efficiency. Luciferase activity was measured by a dual‐luciferase reporter assay system kit (Promega, WI) 48 h after the transfection. Finally, the mean and standard deviation of the relative fluorescence intensity obtained in three independent experiments were calculated.

### 2.11 Animal experiments and histological assessments

All animal experimental procedures were approved by the Institutional Animal Care and Use Committee (IACUC). In the in vivo xenograft experiment, a total of 5×10^5^ KYSE170‐shBACH1, KYSE150‐BACH1, or corresponding control cells were subcutaneously injected into BALB/c nude mice. The tumor size was measured and the volume was calculated at 3‐ or 4‐day intervals. After 4 weeks, the mice were euthanized. The xenograft tumors were weighed and fixed with 4% paraformaldehyde for hematoxylin and eosin (H&E) and immunohistochemical (IHC) staining using antibodies against N‐cadherin (#13116, CST), vimentin (#5741, CST), Slug (#TA800167, OriGene Technologies), CD31 (#ab28364, Abcam), and VEGFC (#sc‐374628, Santa Cruz Biotechnology).

### 2.12 Data mining and statistical analysis

The RNA‐sequencing and clinical data of 95 patients with ESCC in the TCGA database (https://cancergenome.nih.gov/) were used for survival analysis. Detailed OS information was available for 95 ESCC cases, and 76 ESCC cases had detailed DFS information. The follow‐up period was 0 to 68 months. The median of patient follow‐up was 12 months. RNA‐sequencing data were classified into two groups (*BACH1* low and high) based on the cut‐off values obtained from the receiver operating characteristic (ROC) curve. The Gene Expression Omnibus (GEO) (https://www.ncbi.nlm.nih.gov/geo/) accession number GSE23400 dataset, hTF target database (http://bioinfo.life.hust.edu.cn/hTFtarget#!/tf), and JASPAR database (http://jaspar.genereg.net/) are publicly available.

All analyses were performed and visualized using GraphPad Prism 8.0 (GraphPad Software). Two groups were compared using Student's t‐test or Mann‐Whitney U test. The Spearman rank correlation coefficients between the mRNA expression of BACH1 and VEGFC were calculated. Comparison of categorical data was performed by the Chi‐squared test. The relationships between the *BACH1* levels and patient survival were determined by the Kaplan‐Meier method and log‐rank analysis. *P* values <0.05 were considered statistically significant.

## 3 RESULTS

### 3.1 BACH1 is highly expressed in ESCC tissue and predicts poor prognosis

**FIGURE 1:**
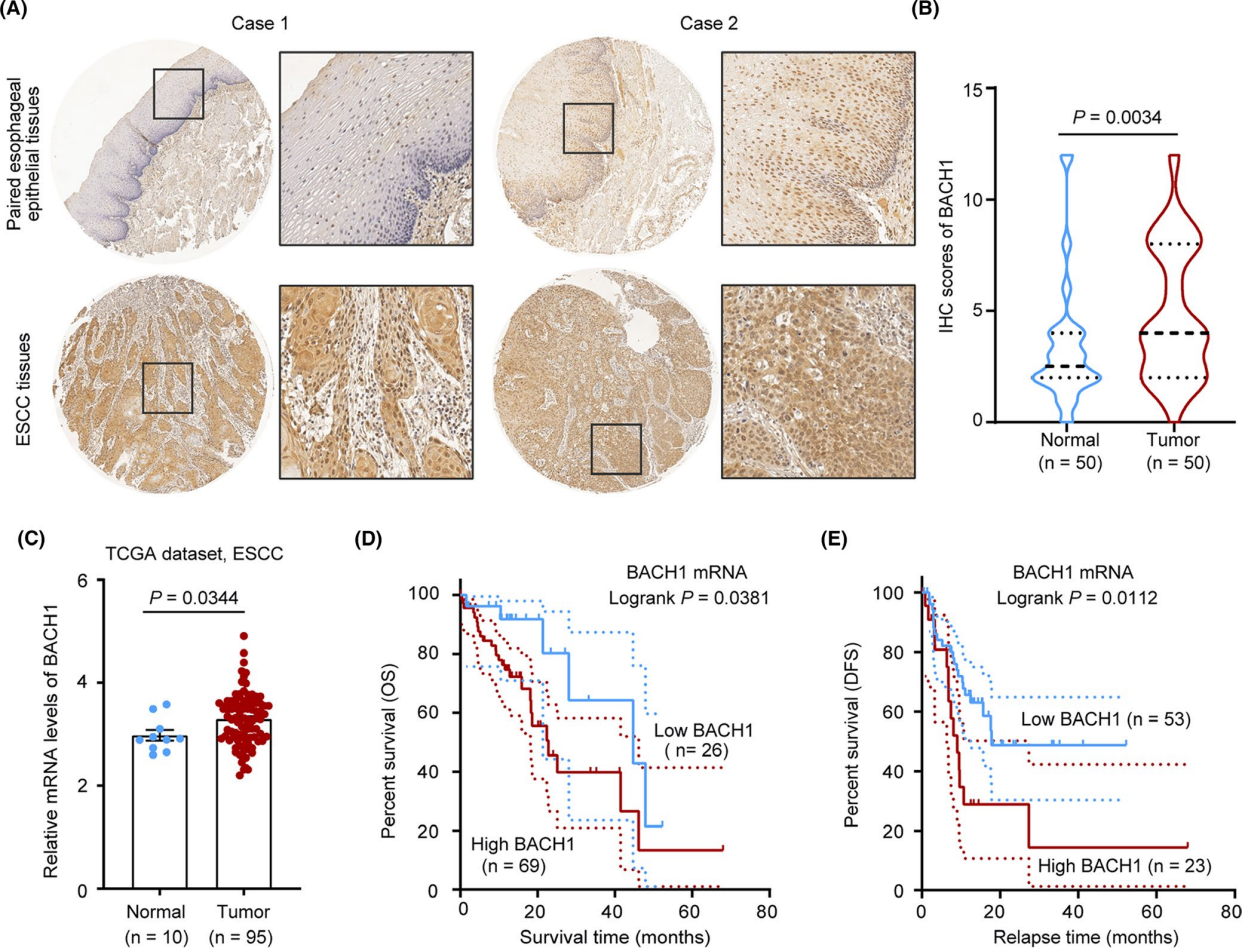
BACH1 is expressed at a high level in ESCC. (A) Representative images of BACH1 expression in paired esophageal epithelial tissues (upper row) and ESCC tissues (lower row) obtained by IHC staining of ESCC tissue arrays. (B) Comparison of the IHC scores of BACH1 in paired esophageal epithelial tissues and ESCC tissues by Mann‐Whitney U test. (C) Comparison of *BACH1* mRNA levels in ESCC tissues and noncancerous tissues in the TCGA database by the Mann‐Whitney U test. (D, E) Kaplan‐Meier plot of the TCGA data showing overall survival (OS) and disease‐free survival (DFS) of ESCC patients with high and low levels of BACH1 assigned based on the cut‐off values obtained by the ROC curve analysis.

To explore the clinical significance of BACH1 in ESCC, we performed an IHC assay to assess the expression of BACH1 in 50 ESCC tissues and paired esophageal epithelia tissues. The expression of BACH1 was weak or moderate in the noncancerous tissues and moderate or strong expressed in the tumor tissues (Figure 1A). Statistical analysis indicated that the IHC score of BACH1 expression in the ESCC tissues was considerably higher than that in the noncancerous tissues (*P* = 0.0034) (Figure 1B). We also analyzed the dataset of the ESCC tissues from the TCGA cohort, and the results indicated that the average expression of *BACH1* mRNA was significantly increased in the ESCC tissues compared with that in the noncancerous tissues (*P* = 0.0344) (Figure 1C). Then, we analyzed the associations between *BACH1* mRNA levels and survival time based on the TCGA‐ESCC database. The results showed that the patients in the high‐level group had either poorer OS (*P* = 0.0385, Figure 1D) or shorter DFS (*P* = 0.0112, Figure 1E) than those of the patients in the low‐level group. Overall, these data suggested that BACH1 may play a crucial role in ESCC progression.

### 3.2 Expression of BACH1 in ESCC cells

**FIGURE 2:**
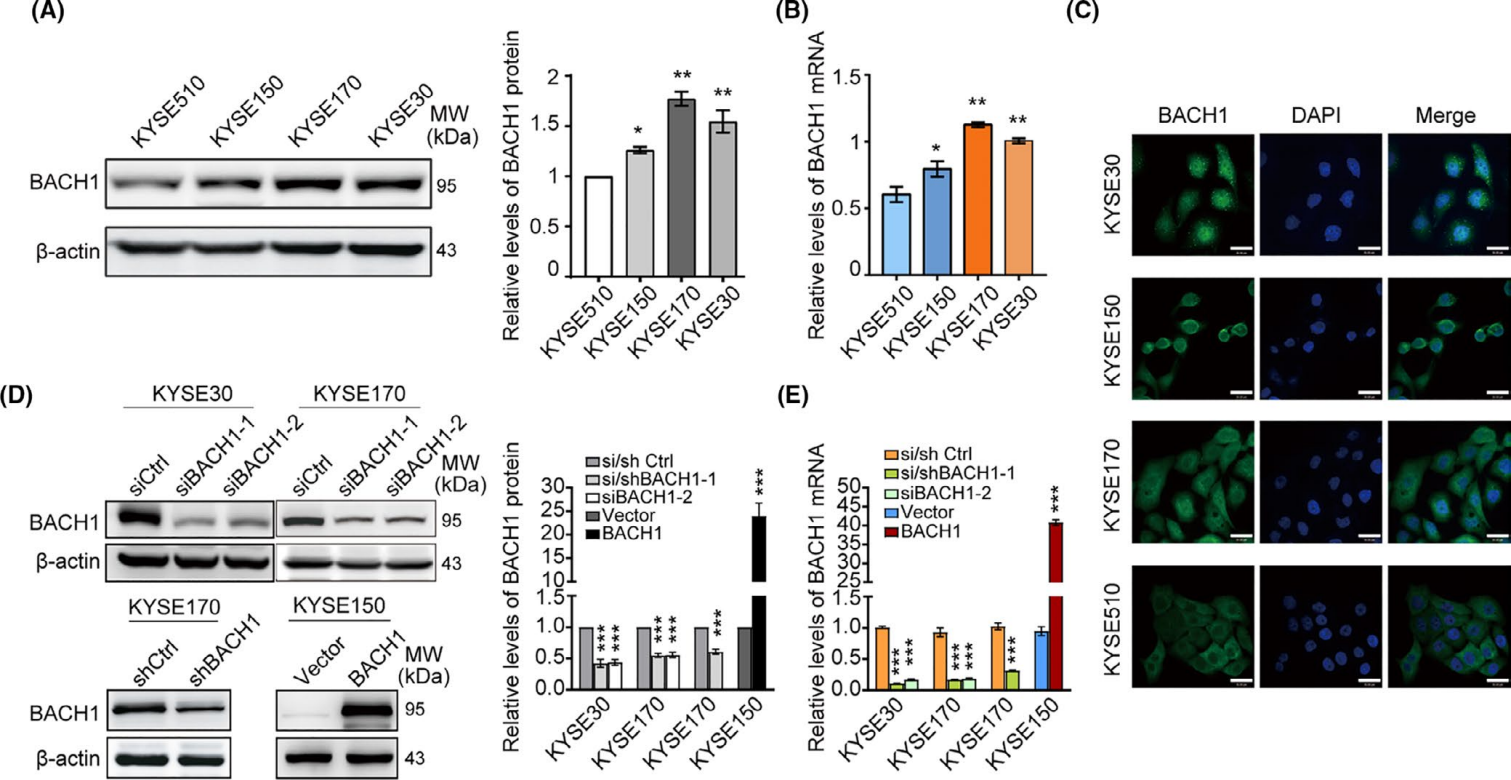
The expression of BACH1 in ESCC cells. (A) Western blot analysis and (B) qPCR analysis of the levels of BACH1 in various ESCC cell lines. (C) Immunofluorescence analysis of BACH1 (green) and DAPI (blue, cell nuclei) in ESCC cell lines. Scale bars, 30 μm. (D) Western blot analysis and (E) qPCR analysis of the expression of *BACH1* in the transient or stable knockdown of BACH1 in KYSE30 and KYSE170 cells and the corresponding control cells and the cells with stable overexpression of BACH1 using KYSE150‐BACH1 and the corresponding KYSE150‐vector control cells. The protein levels of BACH1 were normalized to those of β‐actin, and fold changes are shown relative to the control group. *P < 0.05, **P < 0.01 and ***P < 0.001 by Student's t test.

The expression of BACH1 in four ESCC cell lines was assessed to evaluate the expression profile of BACH1 in vitro. The data of Western blot and qPCR analysis indicated that the expression of BACH1 was relatively higher in KYSE30 and KYSE170 cells and lower in KYSE150 and KYSE510 cells (Figure 2A and 2B), and the data of the IF assays showed that BACH1 was localized in the nucleus and cytoplasm of ESCC cells (Figure 2C). Thus, *BACH1* expression was transiently knocked down in KYSE30 and KYSE170 cells by two siRNA oligos against BACH1. A stable *BACH1* overexpression cell line was generated from KYSE150 cells, and a stable BACH1 knockdown cell line was generated from KYSE170 cells to investigate and validate the long‐term effect of BACH1. The knockdown and overexpression efficiencies of *BACH1* were validated by qPCR and Western blot analysis (Figure 2D and 2E).

### 3.3 BACH1 enhances the migration and invasion of ESCC cells in vitro

**FIGURE 3:**
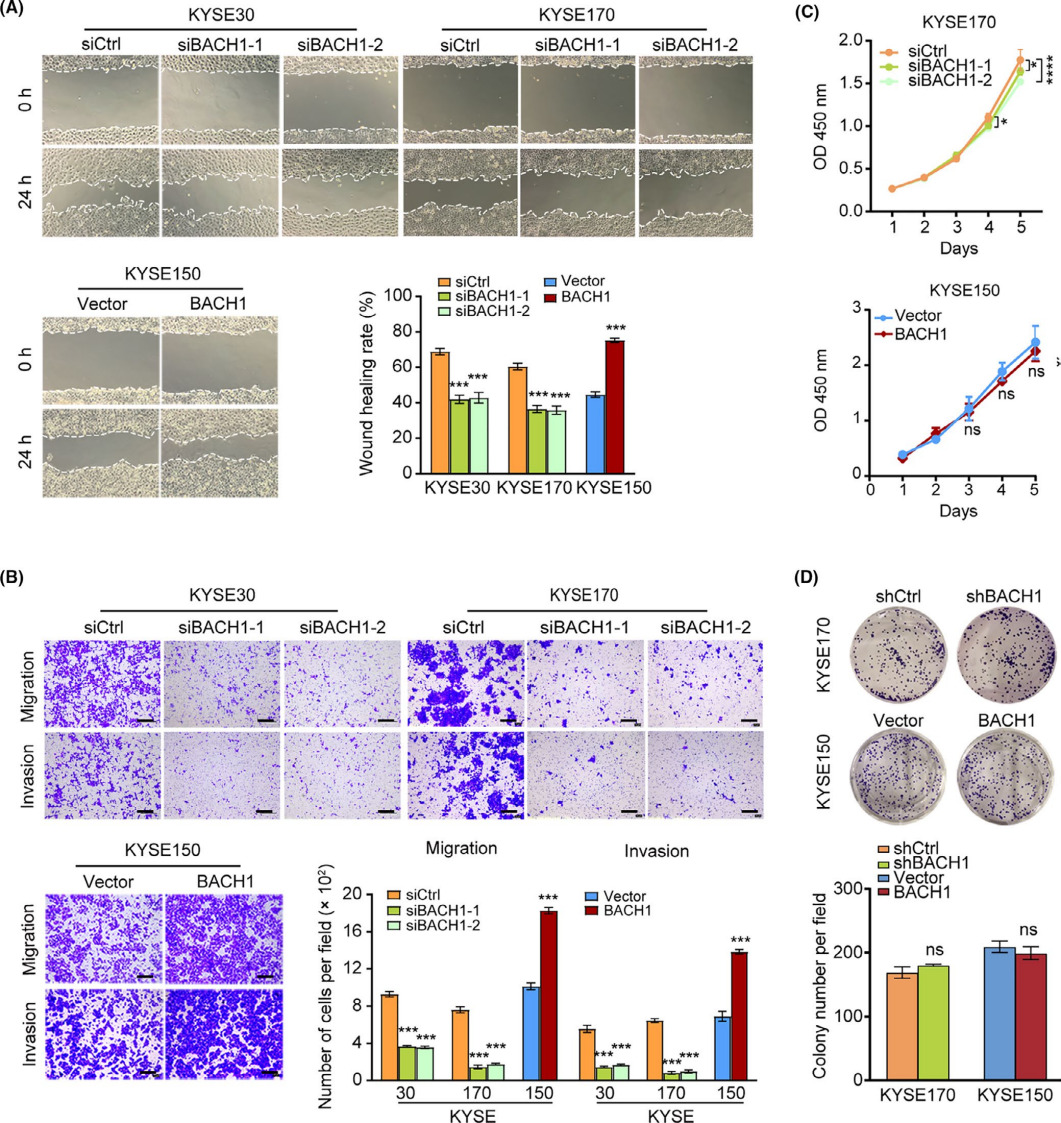
Effects of BACH1 expression on the metastasis‐related properties and proliferation of ESCC cells in vitro. (A) Wound healing assays were performed in KYSE30‐siBACH1‐1/‐2, KYSE170‐siBACH1‐1/‐2, and KYSE150‐BACH1 cells and corresponding control cells cultured for 24 h. (B) Transwell assays were performed in KYSE30‐siBACH1‐1/‐2, KYSE170‐siBACH1‐1/‐2, and KYSE150‐BACH1 cells and corresponding control cells cultured for 24 h. (C) CCK‐8 proliferation assays and (D) colony formation assays in KYSE170‐si/shBACH1 and KYSE150‐BACH1 cells and corresponding control cells. *P < 0.05, **P < 0.01, ***P < 0.001, and ****P < 0.0001 by Student's t test or two‐way ANOVA combined with multiple comparisons test.

To determine the functional role of BACH1 in ESCC, we detected the effect of BACH1 on the invasion and migration abilities of the cells using wound healing and Transwell assays in vitro. The results of the wound healing assays showed that overexpression of *BACH1* dramatically increased the migration of KYSE150 cells, and knockdown of *BACH1* significantly attenuated the migration of KYSE30 and KYSE170 cells (Figure 3A). The results of the Transwell assays indicated that overexpression of *BACH1* notably promoted the migration and invasion of KYSE150 cells, and the opposite effects were observed in *BACH1* knockdown KYSE30 and KYSE170 cells (Figure 3B), confirming that the effect of BACH1 on ESCC cells in vitro is consistent with promotion of invasion and metastasis. We subsequently detected the effect of BACH1 on short‐term and long‐term proliferation using CCK assays and colony formation assays in the cells. Knockdown of *BACH1* in KYSE170 cells inhibited short‐term cell proliferation; however, *BACH1* overexpression did not influence the proliferation of KYSE150 cells (Figure 3C). BACH1 had no effect on long‐term cell growth and survival (Figure 3D). Thus, BACH1 significantly enhanced tumor migration and invasion in ESCC cells.

### 3.4 BACH1 may induce the EMT in ESCC cells

**FIGURE 4:**
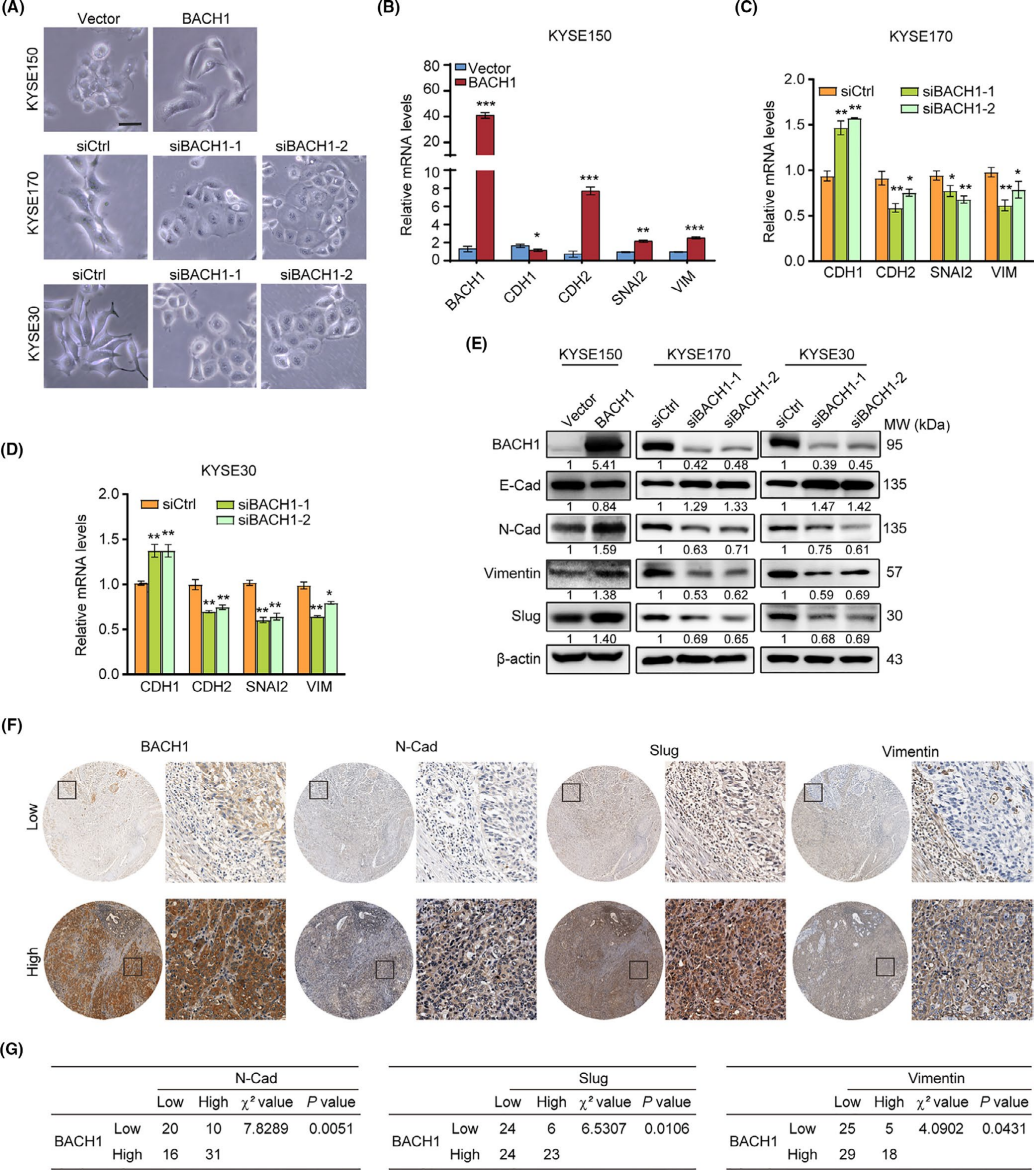
BACH1 promoted metastasis by activating EMT‐related genes. (A) Representative images showing that *BACH1* overexpression in KYSE150 cells and *BACH1* knockdown in KYSE170 and KYSE30 cells using two siRNA oligos (siBACH1‐1 and siBACH1‐2) resulted in a morphological transition compared with the control cells. Scale bar, 20 μm. (B‐D) The mRNA levels of *CDH1*, *CDH2*, *SNAI2*, and *VIM* in *BACH1*‐overexpressing cells (B) and *BACH1*‐knockdown cells (C, D) were quantified by qPCR. (E) Western blot analysis of the EMT markers E‐cadherin, N‐cadherin, vimentin, and Slug in *BACH1*‐overexpressing cells and *BACH1*‐knockdown cells. The values of the ratios under each Western blot image indicate the levels of the expression of EMT markers, which were normalized to the expression of β‐actin and to the corresponding levels in the control group. (F) Representative images of BACH1 expression in ESCC tissues with various expression levels of N‐cad, Slug, and vimentin. (G) Correlation of the expression of N‐cadherin, Slug, and vimentin with BACH1 levels in the ESCC tissues (n = 77); Chi‐squared test was used for comparison. *P < 0.05, **P < 0.01, and ***P < 0.001 by Student's *t* test.

Intriguingly, *BACH1* overexpression and knockdown caused morphological changes in ESCC cells. *BACH1* overexpression resulted in an apparent morphological transition from epithelial to mesenchymal phenotypes in KYSE150‐BACH1 cells, and *BACH1* depletion using two specific siRNAs resulted in significant epithelial morphology, some cells showed cobblestone‐like morphology of KYSE170 and KYSE30 cells (Figure 4A). It reminded us whether BACH1 was involved in the induction of the EMT in ESCC cells.

The expression of mRNAs and some EMT marker proteins, including E‐cadherin, N‐cadherin, vimentin, and Slug, were detected by qPCR and Western blot, respectively, to demonstrate the effect of BACH1 on the EMT. As shown in Figure 4B‐4E, knockdown of *BACH1* suppressed the expression of N‐cadherin, vimentin, and Slug and upregulated the levels of E‐cadherin in KYSE30‐siBACH1 and KYSE170‐siBACH1 cells. Conversely, overexpression of *BACH1* in KYSE150‐BACH1 cells increased the levels of N‐cadherin, vimentin, and Slug and reduced the expression of E‐cadherin, suggesting that BACH1 induced the EMT.

The protein levels of N‐cadherin, Slug, and vimentin were further assayed in 77 ESCC tissue samples by IHC to investigate their correlations with BACH1 (Figure 4F). The results of the Chi‐square test showed that high expression of BACH1 protein was positively associated with high levels of N‐cadherin (*P* = 0.0051), Slug (*P* = 0.0106), and vimentin (*P* = 0.0431) (Figure 4G). Therefore, these results confirmed that BACH1 facilitates the EMT in ESCC.

### 3.5 BACH1 transcriptionally upregulates the expression of *CDH2* in ESCC cells

**FIGURE 5:**
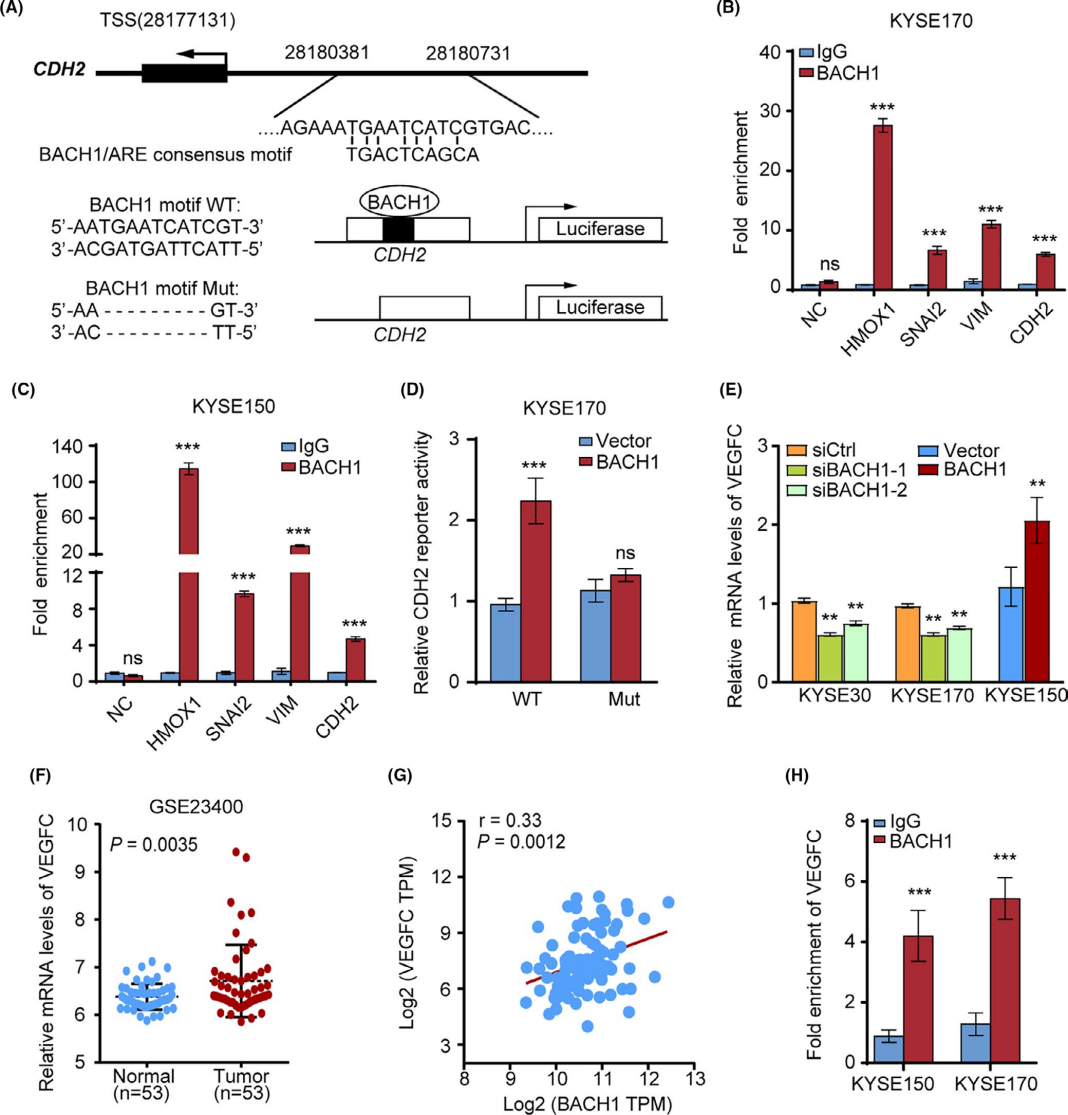
BACH1 transcriptionally activated *CDH2*, *SNAI2*, and *VEGFC* to promote the EMT and angiogenesis. (A) Upper panel, schematic diagram showing the BACH1/antioxidant response element (ARE) consensus motif and predicted BACH1‐binding sequence in the *CDH2* promoter region ~3400 bp upstream of the transcriptional start sites (TSSs). Lower panel, schematic diagram showing 350 bp promoter sequences containing the BACH1‐binding sequence or deletion subcloned into the pGL3 luciferase reporter vector. (B, C) ChIP‐qPCR analysis of BACH1 binding to the promoter regions of *HMOX1*, *SNAI2*, *VIM*, and *CDH2* in (B) KYSE170 and (C) KYSE150 cells (comparison vs. control IgG binding; *HMOX1* was used as a positive control). (D) Luciferase assays of the *CDH2* promoter and the mutant promoter in KYSE170 cells with exogenous overexpression of *BACH1* or cells transfected with an empty vector. (E) The mRNA levels of *VEGFC* in *BACH1*‐knockdown (KYSE170‐siBACH1‐1/‐2 and KYSE30‐siBACH1‐1/‐2) and *BACH1*‐overexpressing (KYSE150‐BACH1) cells were quantified by qPCR. (F) Analysis of *VEGFC* mRNA levels in ESCC tissues and paracancerous tissues based on the GSE23400 dataset. (G) The mRNA expression of *VEGFC* was positively correlated with BACH1 expression according to the analysis of the data of the TCGA database. (H) ChIP‐qPCR analysis of BACH1 binding to the promoter regions of *VEGFC* compared with that for the control IgG binding. **P < 0.01, ***P < 0.001 by Student's *t* test.

Previous studies have reported that BACH1 can directly bind to the promoters of the EMT markers, including *SNAI2* in ovarian cancer^15^ and *FOXA1* in pancreatic cancer.^17^ The ChIP‐seq dataset targeting BACH1 in the hTFtarget database was investigated to determine whether BACH1 binds to the promoters of other EMT markers in ESCC cells. The results based on the ChIP‐seq data from the GEO: GSE31477 dataset demonstrated that BACH1 was bound to the promoter regions of *CDH2*. Then, we downloaded the sequence of the *CDH2* promoter region (−4000/+100) from the UCSC Genome Browser and identified a BACH1‐binding sequence that was located ~3446 base pairs (bp) upstream of the transcriptional start site (TSS) of *CDH2* using the JASPAR database (Figure 5A). ChIP assays were performed in KYSE170 and KYSE150 cells by using an anti‐BACH1 antibody or a control IgG. The results of qPCR confirmed that BACH1 was bound to the *CDH2* promoter. Additionally, BACH1 was able to bind with the promoter regions of *SNAI2* and *VIM*, as expected as the data reported in a previous study^15^ (Figure 5B and 5C).

To determine whether the BACH1 binding site is involved in *CDH2* transcriptional activation, we constructed luciferase reporter vectors with a 350 bp insertion (Chr18:28180381–28180731) of *CDH2* promoter regions containing the BACH1‐binding sequence or a corresponding mutated version with deletion of the BACH1‐binding sequence (Figure 5A). The relative luciferase activity of the *CDH2* wild‐type promoter was twofold higher in *BACH1*‐overexpressing KYSE170 cells than that in KYSE170 cells transfected with a control vector (Figure 5D). However, the relative luciferase activity of the cells transfected with mutated *CDH2* promoters was similar in *BACH1*‐overexpressing and control vector‐transfected KYSE170 cells. These findings confirmed that BACH1 transactivates the promoter of *CDH2* in ESCC cells.

### 3.6 BACH1 upregulates *VEGFC* to promote angiogenesis

Blood vessel formation is important for tumor expansion and metastatic spread.^23^ Previous studies have shown that BACH1 promotes angiogenesis by modulating VEGFC expression in ovarian cancer and colorectal cancer.^10^, ^16^ Thus, we investigated the correlation of BACH1 with VEGFC in ESCC. Initially, we detected a decrease in the levels of mRNA of *VEGFC* in KYSE170‐siBACH1 and KYSE30‐siBACH1 cells with knocked down *BACH1* expression (Figure 5E). In contrast, enhanced *BACH1* expression was associated with an increase in the level of mRNA of *VEGFC* in KYSE150‐BACH1 cells (Figure 5F). Then, we assessed the expression of *VEGFC* in ESCC using GEO data (GSE23400). The expression of mRNA of *VEGFC* was dramatically upregulated in the ESCC tissues compared with that in the noncancerous tissues (Figure 5B). Spearman correlation analysis of the TCGA‐ESCC database showed that the expression of *VEGFC* mRNA was positively associated with the expression of *BACH1* mRNA (Figure 5G). Furthermore, consistent with the data of a previous study,^16^ the results of ChIP‐qPCR of KYSE150 and KYSE170 cells showed that BACH1 was able to bind to the promoter region of *VEGFC* to regulate the expression of VEGFC (Figure 5H). Thus, BACH1 was able to promote metastasis partially by upregulating the transcription of *VEGFC* and facilitating angiogenesis.

### 3.7 BACH1 facilitates the proliferation of ESCC cells and angiogenesis in vivo

**FIGURE 6:**
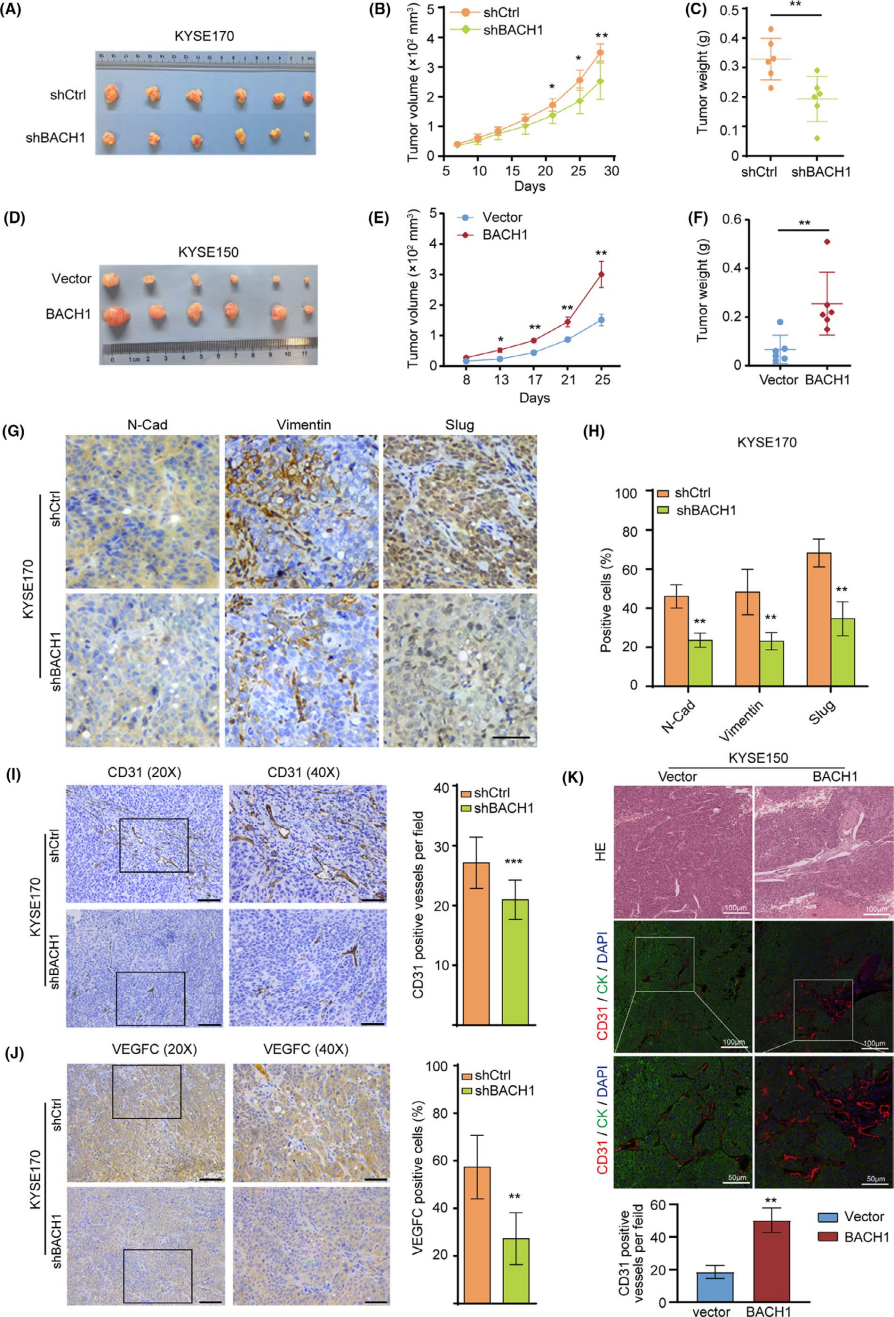
BACH1 promoted in vivo cell growth and angiogenesis in ESCC. (A‐C) KYSE170‐shBACH1 and KYSE170‐shCtrl cells were subcutaneously injected into the flanks of nude mice (n = 6). The xenograft tumors were obtained after 4 weeks, and (B) the tumor volume and (C) weight were measured. (D‐F) KYSE150‐BACH1 and empty vector control cells were subcutaneously injected into the flanks of nude mice (n = 6). The xenograft tumors were obtained after 4 weeks, and (E) the tumor volume and (F) weight were measured. (G‐H) Immunohistochemical staining for N‐cadherin, vimentin, and Slug in xenograft tumors derived from KYSE170‐shBACH1 and KYSE170‐shCtrl cells. Scale bar (40×), 50 μm. (I, J) Immunohistochemical staining for (I) CD31 and (J) VEGFC in xenograft tumors derived from KYSE170‐shBACH1 and KYSE170‐shCtrl cells. Scale bars (20×), 200 μm, scale bars (40×), 50 μm. (K) HE and immunofluorescence staining for CD31 in xenograft tumors derived from KYSE150‐BACH1 and KYSE150‐vector cells. Scale bars (20×), 100 μm, scale bars (40×), 50 μm. *P < 0.05, **P < 0.01, and ***P < 0.001 by Student's t test.

Subsequently, we detected the long‐term effect of BACH1 on tumor progression in vivo. A xenograft model was generated by subcutaneous injection of KYSE170‐shBACH1/KYSE170‐shCtrl and KYSE150‐BACH1/KYSE150‐vector cells into the flanks of nude mice, and xenograft tumors were resected and harvested. The results showed that knockdown of *BACH1* significantly attenuated the tumor growth because the tumors derived from KYSE170‐shBACH1 cells were smaller than the tumors derived from the control cells (*P* < 0.01) (Figure 6A‐6C); moreover, overexpression of BACH1 enhanced the tumor growth because the tumors derived from KYSE150‐BACH1 cells were larger than the tumors derived from the control cells (*P* < 0.01) (Figure 6D‐6F). Then, we examined the expression of EMT markers in vivo. The results of immunohistochemical staining (IHC) of xenograft tumors derived from KYSE170‐shBACH1 and KYSE170‐shCtrl cells showed that *BACH1* knockdown was accompanied by low expression of N‐cadherin, vimentin, and Slug in vivo (Figure 6G and 6H).

Additionally, we performed immunohistochemical and IF staining for vascular endothelial markers CD31 and VEGFC in xenograft tumors to evaluate angiogenesis. The results showed that BACH1 knockdown significantly inhibited the density of CD31^+^ blood vessels and VEGFC expression in xenograft tumor tissues derived from mice subcutaneously injected with KYSE170‐shBACH1 cells compared with those derived from mice injected with control KYSE170‐shCtrl cells (Figure 6I and 6J). However, BACH1 overexpression significantly increased the density of CD31^+^ blood vessels in xenograft tumor tissues derived from mice subcutaneously injected with KYSE150‐BACH1 cells compared with that derived from mice injected with KYSE150‐Vector control cells (Figure 6K). Overall, these results suggested that BACH1 enhanced tumor growth in a xenograft model partially by facilitating angiogenesis and the EMT.

## 4 DISCUSSION

ESCC is the most common histological type of esophageal cancer in eastern Asia and north central China, accounting for approximately 90% of all esophageal cancers.^24^ ESCC has a high rate of cancer‐related mortality.^25^ Due to strong invasive and metastatic abilities of ESCC, most patients have an advanced stage of the disease with deep invasion of muscle layers, lymph node metastasis, or nearby organ metastasis at first diagnosis, subsequently resulting in a poor outcome. Hence, identification of regulatory molecules involved in the progression and metastasis of ESCC may provide potential therapeutic targets or biomarkers. Increasing evidence indicated that BACH1 promotes metastasis in various human cancers; however, the functional role and mechanism of action of BACH1 in ESCC have not been elucidated. Therefore, we investigated whether alterations in *BACH1* expression influence the metastasis of ESCC and determined potential mechanism of action of BACH1.

The results of the present study indicated that BACH1 promoted the migration and invasion of ESCC cells in vitro and that silencing *BACH1* attenuated the growth of xenograft tumors in vivo. Moreover, BACH1 expression was upregulated in the ESCC tissues compared with that in the paired noncancerous tissues according to the data of IHC assay. Notably, a high level of *BACH1* was significantly associated with poor OS and DFS according to the TCGA datasets. Unfortunately, correlations between BACH1 expression and TNM stage were not detected in the present study (Table S3), which can be attributable to many reasons, such as the impact of the tumor microenvironment, tumor heterogeneity, and the limited number of cases; thus, this observation requires confirmation in a larger cohort. These results suggested that BACH1 may be an important regulator of ESCC progression.

Tumor cells that undergo the EMT lose cell–cell adhesion and polarity and acquire mesenchymal characteristics with stronger migration and invasion properties.^26^ Accumulating recent evidence indicated that the EMT is common in tumor cells during tumor progression and correlates with local invasiveness and metastatic potential of various tumors, including ESCC.^15^, ^17^, ^27^, ^28^, ^29^ Morphological changes caused by BACH1 were observed in the present study; *BACH1* overexpression increased the expression of N‐cadherin, vimentin, and Slug and decreased the level of the epithelial marker E‐cadherin, and knockdown of *BACH1* showed the opposite effects. Consistent associations of the expression of BACH1 and the levels of N‐cadherin, Slug, and vimentin were observed in clinical samples, confirming that BACH1 promotes ESCC progression partially by inducing the EMT.

Several studies have reported that BACH1 contributes to transcriptional regulation as a transcriptional activator or suppressor. BACH1 was able to activate the transcription of metastasis‐related genes,^9^ metabolism‐related genes,^19^ and EMT‐related genes.^15^ The results of the present study confirmed the binding of BACH1 with the promoters of *SNAI2* and *VIM* in ESCC cells. BACH1 directly binds to the *CDH2* promoter to stimulate the transcriptional activity of *CDH2* according to the results of dual‐luciferase reporter and ChIP‐qPCR assays. N‐cadherin is a key mesenchymal marker that promotes tumor metastasis and mobility by directly mediating cell–cell adhesion, activating the expression of matrix metalloproteinase‐9 (MMP9), and stimulating the activity of fibroblast growth factor receptor (FGFR).^30^, ^31^, ^32^ Previous studies have shown that overexpression of N‐cadherin in epithelial cancer cells promotes metastasis in bladder cancer,^33^ prostate cancer,^34^ pancreatic cancer,^35^ melanoma,^36^, ^37^ and thyroid cancer.^38^ Our study provided new evidences by which BACH1 induces the EMT to increase the capability of migration and invasion of ESCC cells.

Angiogenesis refers to the formation of new blood vessels from preexisting capillaries or posterior capillaries and is a key step in the rapid growth and distal metastasis of many solid tumors.^39^ Previous studies reported that BACH1 induces angiogenesis and lymphangiogenesis by regulating the transcription of *VEGFC* to promote the expansion and aggressiveness of ovarian carcinoma.^16^ VEGFC is a member of the VEGF family acting via the VEGFR‐3 and VEGFR‐2 receptors. Induction of lymphatic development and remodeling is the major function of VEGFC, and several studies reported that VEGFC also induces angiogenesis via the VEGFC/VEGFR2 or VEGFC/VEGFCR3 axis.^40^, ^41^, ^42^ Additionally, VEGFC was shown to be involved in tumor proliferation, invasion, and metastasis.^43^, ^44^, ^45^ The results of the analysis of the GEO dataset performed in the present study indicated that *VEGFC* was significantly upregulated in ESCC tissues. Inhibition of *BACH1* expression markedly repressed the expression of *VEGFC* in ESCC cells both in vitro and in vivo, attenuated the density of CD31^+^ blood vessels, and reduced angiogenesis in tumor xenografts in agreement with the results of the previous study that demonstrated that BACH1 significantly promotes tumor growth and angiogenesis in colorectal cancer.^10^ The binding of BACH1 to the promoter regions of *VEGFC* was also validated by ChIP‐qPCR assays in ESCC cells in the present study. These data indicated that BACH1 promotes ESCC progression partially by upregulating the transcription of *VEGFC* and facilitating angiogenesis. Notably, recent studies have shown that VEGFC is presumably involved in the induction of the EMT, and high VEGFC expression downregulates E‐cadherin and upregulates N‐cadherin and vimentin.^46^, ^47^, ^48^ Possible contribution of VEGFC to BACH1‐induced EMT suggested in the present study requires additional investigation.

In conclusion, this study revealed essential prometastatic, proangiopoietic, and prognostic roles of BACH1 in ESCC. BACH1 enhanced the transcription of EMT‐related genes *CDH2*, *VIM*, *SNAI2*, and *VEGFC* by binding to the transcription promoter regions of these genes to promote ESCC metastasis and angiogenesis. Our findings demonstrated that BACH1 may be a potential therapeutic target and prognostic biomarker in ESCC.

## CONFLICT OF INTEREST

No conflict of interest to declare.

## ETHICAL APPROVAL

This study was approved by the Institutional Review Board of the Ethics Committee of Cancer Hospital, Chinese Academy of Medical Sciences (ID: NCC1783) and performed in accordance with the guidelines of the Declaration of Helsinki.

## Supporting information

Table S1‐S3

## Data Availability

The data presented in this study are available from the corresponding authors on request.
