# Supplementary material for: BACH1 promotes the progression of esophageal squamous cell carcinoma by inducing the epithelial–mesenchymal transition and angiogenesis
Source: Cancer Med. 2021 May 1;10(10):3413–26. doi: 10.1002/cam4.3884 (PMC8124123; doi:10.1002/cam4.3884)
Supplement: Supplementary file 1 — Table S1‐S3 [file CAM4-10-3413-s001.docx]

**Appendix**

**Supplementary Table S1.** Primer sequences for qPCR

| Primer name | Sequence (5'-3') |
| --- | --- |
| *BACH1-*F | AGACGACTCTGAGACGGACA |
| *BACH1-*R | CGCTGTGCAGCAATTCTGTT |
| *CDH1-*F | ACACTGGTGCCATTTCCACT |
| *CDH1-*R | TTAGGGCTGTGTACGTGCTG |
| *CDH2-*F | CAGTATCCGGTCCGATCTGC |
| *CDH2-*R | GAGCTGTGGGGTCATTGTCA |
| *SNAI2-*F | CCAAGCTTTCAGACCCCCAT |
| *SNAI2-*R | TGCAGCTGCTTATGTTTGGC |
| *VIM*-F | AAATGGCTCGTCACCTTCGT |
| *VIM*-R | CAGCTTCCTGTAGGTGGCAA |
| *VEGFC*-F | CAGTGCCTCTCTCTCAAGGC |
| *VEGFC*-R | GCTGGCAGGGAACGTCTAAT |
| *GAPDH*-F | GGAGCGAGATCCCTCCAAAAT |
| *GAPDH*-R | GGCTGTTGTCATACTTCTCATGG |

**Supplementary Table S2.** Primer sequences for ChIP-qPCR

| Primer name | Sequence (5'-3') |
| --- | --- |
| *HMOX1*-F | AGTCGCGATTTCCTCATCCC |
| *HMOX1*-R | TTCCCTTTGTTTCCGCGAGT |
| *SNAI2*-F | ACGCAAGGTAGATGGCCCAC |
| *SNAI2*-R | GTACCCTGCAGAGGCAGTCT |
| *VIM*-F | GATCCTCAACCTCCTCTGCAGG |
| *VIM*-R | CGGGCAGTCTGGTAGACACT |
| *CDH2*-F | CCTGCCTGACAAAACACACC |
| *CDH2*-R | TCTGAACACATGGCCCGTTT |
| *VEGFC*-F | CCGAGTCTGATGGGATGGAA |
| *VEGFC*-R | GCCTTTGTTGATACAGCCTTGG |
| Negative control-F | TACCGCGGACCAAAGATGAA |
| Negative control-R | ATTTTGCTGCAGCCCGC |

**Supplementary Table S3.** Correlations between BACH1 expression and clinical and pathological characteristics

| Patient characteristics (n) | BACH1 expression | | *P-*value^a^ |
| --- | --- | --- | --- |
|  | Positive (n= 19) | Negative (n=31) |  |
| Gender |  |  | 0.4085 |
| Male (35) | 12 | 23 |  |
| Female (15) | 7 | 8 |  |
| Age (years) |  |  | 0.3413 |
| >=60 (20) | 6 | 14 |  |
| <60 (30) | 13 | 17 |  |
| Histological grade | |  | 0.2202 |
| I (8) | 5 | 3 |  |
| II (33) | 10 | 23 |  |
| III (9) | 4 | 5 |  |
| T stage |  |  | 0.3912 |
| T1 (1) | 0 | 1 |  |
| T2 (9) | 4 | 5 |  |
| T3 (32) | 10 | 22 |  |
| T4 (1) | 1 | 0 |  |
| Unknown (7) | 4 | 3 |  |
| Lymph nodes metastasis | |  | 0.3141 |
| N0 (26) | 12 | 14 |  |
| N1-3 (22) | 7 | 15 |  |
| Unknown (2) | 0 | 2 |  |
| Distal metastasis | |  | 0.2854 |
| Yes (5) | 3 | 2 |  |
| No (45) | 16 | 29 |  |
| AJCC staging |  |  | 0.4907 |
| I~II (19) | 6 | 13 |  |
| III~IV (24) | 9 | 15 |  |
| Unknown (7) | 4 | 3 |  |

^a^ according to chi-squared test.
